# Retraction: Zhao et al. Protective Effect of Glycyrrhizic Acid on Alcoholic Liver Injury in Rats by Modulating Lipid Metabolism. *Molecules* 2018, *23*(7), 1623

**DOI:** 10.3390/molecules24050969

**Published:** 2019-03-09

**Authors:** Xiaowei Huo, Sa Yang, Xiaoke Sun, Xiangbo Meng, Yanyan Zhao

**Affiliations:** Key Laboratory of Pharmaceutical Quality Control of Hebei Province, College of Pharmaceutical Science, Hebei University, Baoding 071002, China; huoxiaoweiforever@163.com (X.H.); mmliu.bio@gmail.com (S.Y.); liuermengmeng@sina.com (X.S.); liuermengmeng@hotmail.com (X.M.)

As the authors of the title paper [1], it is with great regret that we inform the readership of *Molecules* that we have asked the journal’s publisher, MDPI, to retract our paper from the scientific literature. Our reason for this action is that in subsequent work we have obtained results inconsistent with the data published therein and therefore wish to retract this article, pending additional experiments to clarify the discrepancies. In particular, in Figure 1C, for weight increase (g) in groups N, M, P, G1−G7 values of 133 ± 34, 125 ± 39, 126 ± 41, 130 ± 47, 132 ± 36, 127 ± 45, 127 ± 43, 130 ± 37, 131 ± 41 and 128 ± 56, respectively, have since been obtained but there was no significant difference between the weight increase in the model group (M) and normal group (N), which was inconsistent with the previous work. Moreover, for serum TC (Figure 3A in [1]) for groups N, M, P, G1−G7 values of 1.55 ± 0.65, 2.1 ± 0.77 **, 1.49 ± 0.54 ^##^, 1.61 ± 0.63 ^##^, 1.92 ± 0.82, 2.0 ± 0.71, 1.98 ± 0.88, 1.83 ± 0.84, 1.89 ± 0.66, and 1.95 ± 0.94, respectively (** *p* < 0.01 vs. normal group, ^##^ *p* < 0.01 vs. model group), have been obtained, i.e., values that are completely different from the reported work. Finally, for serum LDL (Figure 3D in [1]) values of 0.62 ± 0.34, 0.81 ± 0.32, 0.82 ± 0.48, 0.75 ± 0.37, 0.79 ± 0.47, 0.81 ± 0.57, 0.61 ± 0.37, 0.87 ± 0.31, 0.66 ± 0.23 and 0.58 ± 0.33, respectively, have now been detected, however there was no significant difference between any of the groups, which was not consistent with the previous work. We wish to apologize to MDPI and of course to the wider scientific community for any inconvenience this action may cause. 

The new data in our further study that differs from the data in this paper is concluded below.

## Figures and Tables

**Figure 1C:**
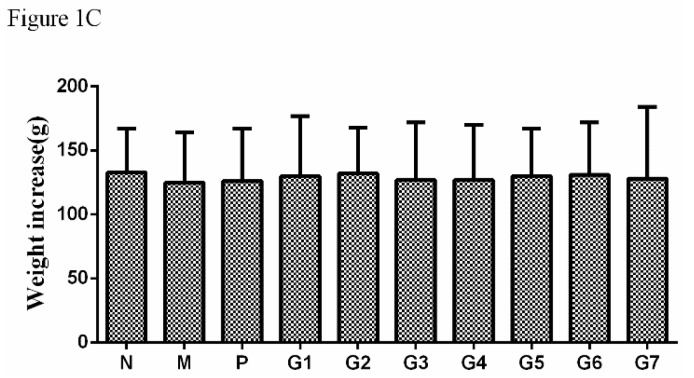
The effect of glycyrrhizic acid (GA) on body weight (Figure 1C). N: normal group, M: model group, P: silybin positive group, G1:18α-GA:18β-GA = 10:0, G2:18α-GA:18β-GA = 8:2, G3:18α-GA:18β-GA = 6:4, G4:18α-GA:18β-GA = 5:5, G5:18α-GA:18β-GA = 4:6, G6:18α-GA:18β-GA = 2:8, G7:18α-GA:18β-GA = 0:10. The values were expressed as mean ± SD (*n* = 8).

**Figure 3:**
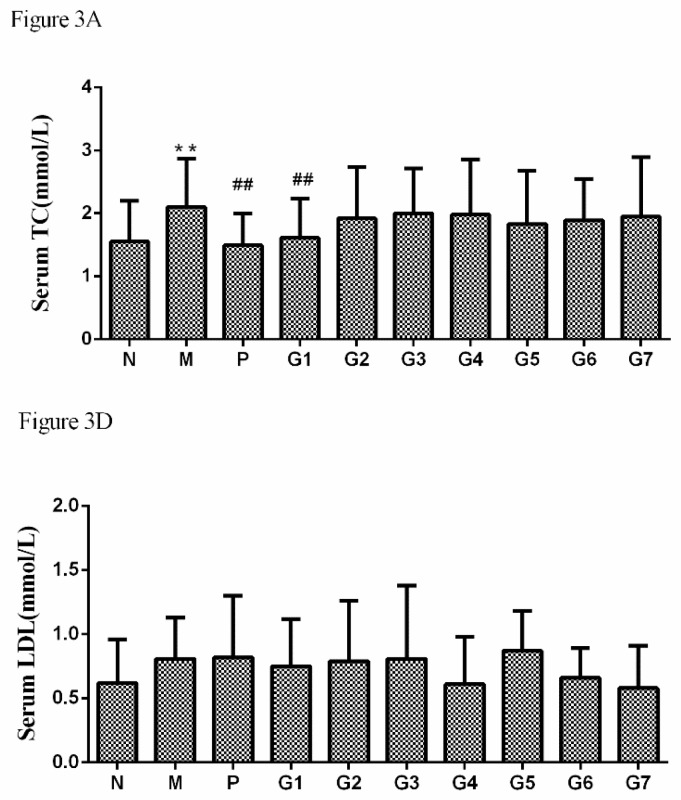
The effect of glycyrrhizic acid (GA) on lipid profile, including Serum TC (A), and Serum LDL (D). N: normal group, M: model group, P: silybin positive group, G1:18α-GA:18β-GA = 10:0, G2:18α-GA:18β-GA = 8:2, G3:18α-GA:18β-GA = 6:4, G4:18α-GA:18β-GA = 5:5, G5:18α-GA:18β-GA = 4:6, G6:18α-GA:18β-GA = 2:8, G7:18α-GA:18β-GA = 0:10. The values were expressed as mean ± SD (*n* = 8). ** *p* < 0.01 vs. normal group, ^##^ *p* < 0.01 vs. model group.
